# Myasthenia gravis complicated with pulmonary infection by *Nocardia cyriacigeorgica*: a case report and literature review

**DOI:** 10.3389/fmed.2024.1423895

**Published:** 2024-10-02

**Authors:** Huifen Zuo, Jiaqing Ye, Chenfei Li, Shijie Li, Jingxin Gu, Na Dong, Yihan Zhao, Jiahao Hao, Minghui Song, Yumei Guo, Weili Gao, Zhenjun Zhao, Lijie Zhang

**Affiliations:** ^1^Department of Clinical Laboratory, Hebei Yiling Hospital, Shijiazhuang, China; ^2^Department of Clinical Laboratory, Hebei Medical University Third Hospital, Shijiazhuang, China; ^3^Department of Orthopedics, Hebei Medical University Third Hospital, Shijiazhuang, China; ^4^Department of Myasthenia Gravis, Hebei Yiling Hospital, Shijiazhuang, China; ^5^Hebei Key Laboratory of Intractable Pathogens, Shijiazhuang Center for Disease Control and Prevention, Shijiazhuang, China

**Keywords:** *Nocardia cyriacigeorgica*, myasthenia gravis, pulmonary infection, trimethoprim-sulfamethoxazole, immunosuppression

## Abstract

Myasthenia gravis (MG) is an autoimmune disease. Patients with MG due to compromised autoimmune regulation, progressive muscle weakness, and prolonged use of immunosuppressants and glucocorticoid, often present with concomitant infections. However, cases of MG complicated by *Nocardia* infection are rare. In this case, we report MG complicated with pulmonary infection by *Nocardia cyriacigeorgica*. A 71-year-old male farmer who was admitted for management of MG. After 7 weeks of treatment of MG, the patient reported improvement. However, clinical presentation, inflammatory markers, and imaging findings supported a diagnosis of pulmonary infection. To further elucidate the etiology, *Nocardia* was identified in sputum smear microscopy and sputum culture, with 16S rRNA gene sequencing confirming *N. cyriacigeorgica*. The patient was prescribed trimethoprim-sulfamethoxazole. After 1 month of treatment, clinical symptoms of MG and pulmonary nocardiosis showed significant improvement. Additionally, we searched PubMed for case reports of *Nocardia cyriacigeorgica* pulmonary infection from 2010 to 2024 and conducted a statistical analysis of the case information. This report aims to highlights the increased risk of pulmonary Nocardia infection in MG patients after the use of steroids and immunosuppressants, thereby enhancing clinical awareness.

## Introduction

Myasthenia gravis (MG) is an autoimmune disease characterized by neuromuscular junction transmission impairment mediated by autoantibodies. *Nocardia* spp. are commonly found in soil, rotten plants and dust particles. Nocardiosis most commonly presents as primary skin infections, pulmonary infections, and disseminated infections (1). *Nocardia* infections are predominantly observed in immunocompromised individuals or those on long-term immunosuppressive therapy (2), also in patients with underlying lung disease (3). Patients with MG due to compromised autoimmune regulation, progressive muscle weakness, and prolonged use of immunosuppressants and glucocorticoids, often present with concomitant infections. Muralidhar Reddy et al. reviewed 21 cases of MG patients with concurrent nocardiosis (4). Until now, only one case of pulmonary nocardiosis caused by *N. cyriacigeorgica* has been identified in a patient with MG at the species level (5).

## Case presentation

A 71-year-old male farmer from Hebei, China, presented with left upper eyelid ptosis in July 2021. He sought medical attention at a local hospital where the edrophonium test yielded positive results. The symptoms fluctuated while receiving treatment with acetylcholinesterase inhibitors. Gradually, weakness in the limbs and difficulty swallowing emerged. In September 2021, further consultation was sought at Hebei Yiling Hospital. The clinical manifestations included bilateral ptosis of the upper eyelids; weakness and easy fatigue of the muscles in the face, neck and limbs; and shortness of breath, with symptoms worsening after activity. The absolute score for myasthenia gravis (MG) was 31 points. Repetitive nerve stimulation demonstrates a wave amplitude decrement >10% in the accessory nerve and bilateral facial nerves. Admitted on September 2nd for MG, with a medical history including right eye enucleation and coronary artery stent placement (regularly taking aspirin enteric-coated tablets, isosorbide dinitrate tablets, and metoprolol tartrate tablets).

Upon hospital admission, arterial blood gas analysis revealed a respiratory acidosis concomitant with metabolic alkalosis (pH 7.39, PO2 40.0 mmHg, PCO2 52.0 mmHg, HCO3 31.5 mmol/L). Some indicators in the blood routine were slightly elevated (WBC 7.53 × 10^9/L, NEUT% 75.8). Additionally, the patient exhibited elevated blood glucose levels (2-h postprandial blood glucose >11 mmol/L). In summary, the patient was treated diabetes with repaglinide tablets (1 mg, tid). For the treatment of MG, in September 2021, acetylcholinesterase inhibitor pyridostigmine bromide (60 mg, qid), glucocorticoid methylprednisolone tablets (20 mg, qd), and immunosuppressants tacrolimus and cyclophosphamide (tacrolimus capsules 2 mg qd AM, followed by 1 mg qd PM. IV infusion of 500 mL 0.9% NaCl with cyclophosphamide 0.4 g, biw). Concurrent traditional Chinese medicine can enhance the therapeutic effects. The efficacy of traditional Chinese medicine mainly lies in enhancing the body's yang energy through warming and tonifying methods, while simultaneously regulating the meridians to promote the circulation of qi and blood, supplementing nutrients or energy, and regulating the extraordinary meridians. The main components of the traditional Chinese medicine are Astragalus, Ginseng, Ophiopogon, Schisandra, Reishi Mushroom, Angelica, Deer Antler, Dodder Seed, Cistanche, Morinda, Platycodon, and Cimicifuga. After 7 weeks of treatment, the patient reported slight improvement in bilateral upper eyelid ptosis, reduced fatigue during swallowing, alleviated limb weakness, less neck fatigue, and improved shortness of breath than before.

**Figure 1:**
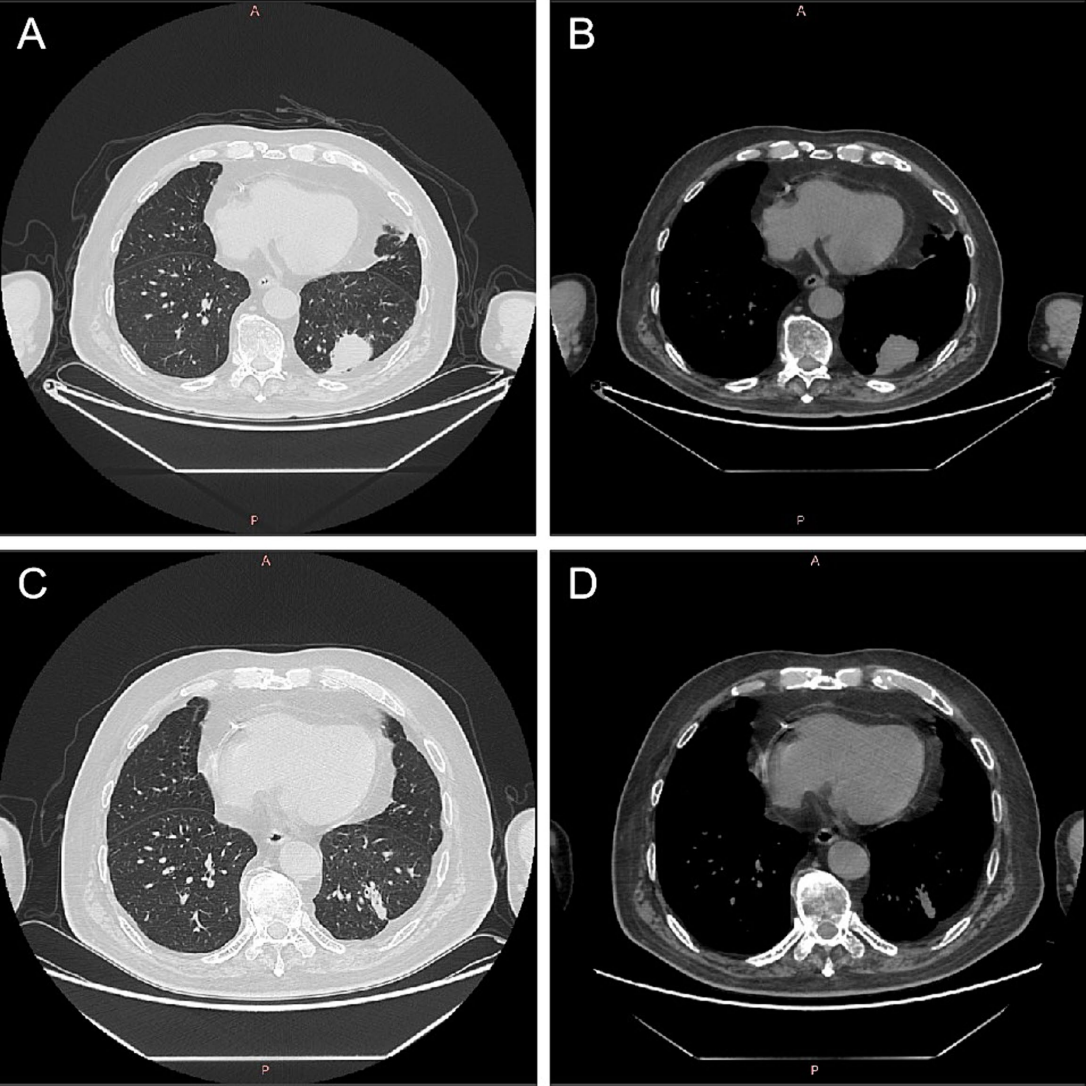
Computed tomography images of lungs before and after *Nocardia* treatment. After 2 months of treatment for myasthenia gravis, there was localized consolidation in the basal segment of the left lower lobe of the lung **(A,B)**. Following 1 month of treatment with trimethoprim–sulfamethoxazole for *Nocardia cyriacigeorgica*, chest CT showed a localized solid lesion in the basal segment of the lower lobe of the left lung, which was less extensive than before **(C,D)**. CT, computed tomography.

**Figure 2:**
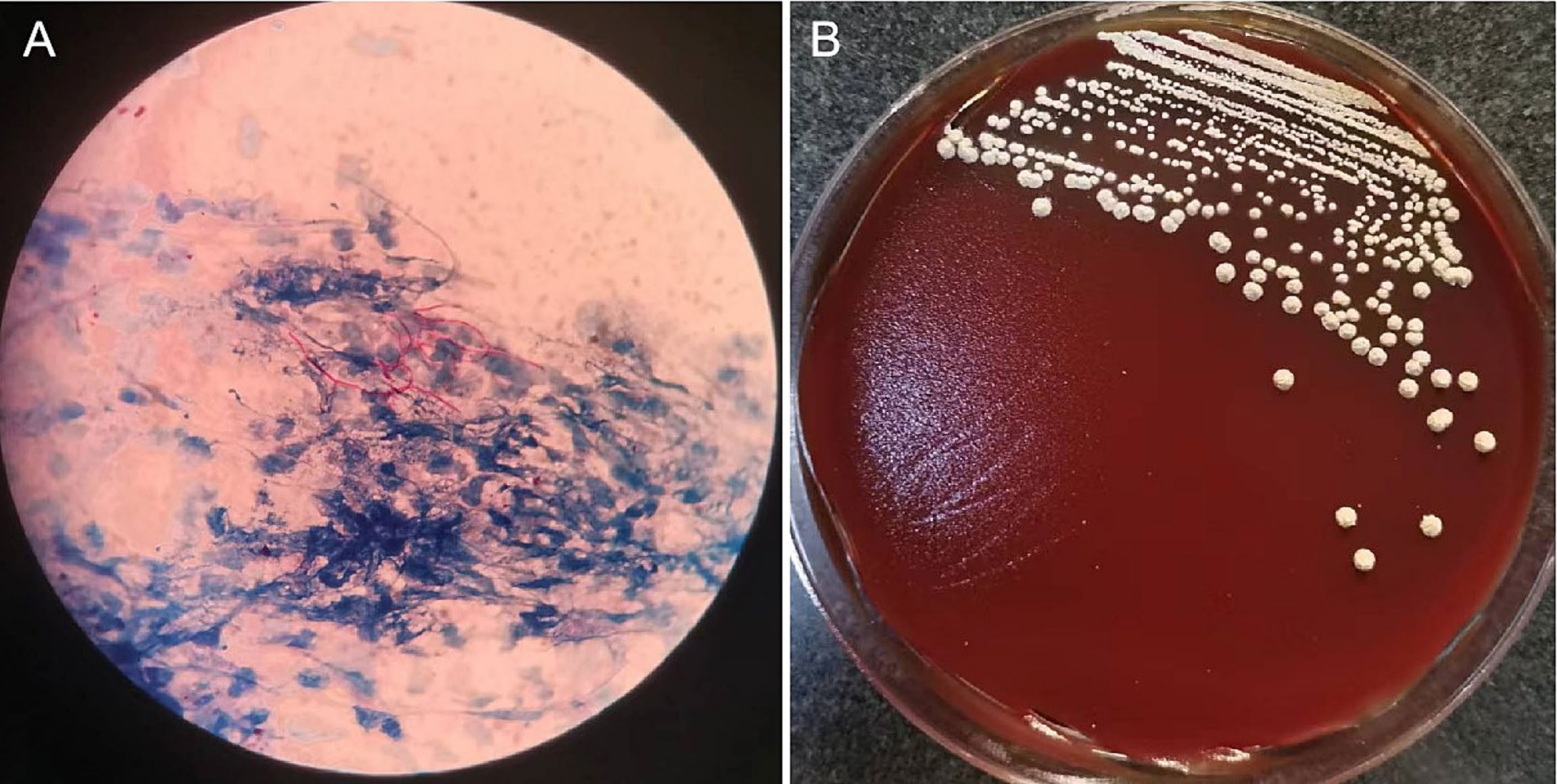
Microscopic pictures of *Nocardia* and colonies isolated from sputum sample.The sputum sample revealed filamentous rods with weak acid-fast staining **(A)**. On Colombia blood agar, grown at 37°C for 5 days, the colonies become wrinkled and stacked like leather, with velvety aerial mycelia on the surface **(B)**.

On October 23, 2021, the patient presented with coughing, sputum production, nasal congestion, and rhinorrhea. Monitoring revealed blood glucose level of 18.2 mmol/L, and elevated inflammatory markers in blood routine (WBC 13.09 × 10^9/L, NEUT% 93.5, CRP 120.7 mg/L). Chest CT showed newly developed localized consolidation in the basal segment of the left lower lobe (Figures 1A,B). Clinical symptoms, inflammatory markers, and imaging support the diagnosis of pulmonary infection. Treatment with ceftriaxone sodium/tazobactam sodium for 5 days showed unsatisfactory results. To further clarify the etiology, filamentous rods with positive weak acid-fast staining were found in 3 days' consecutive sputum smears (Figure 2A), highly suggestive of *Nocardia*. White candida growth was observed in microbial culture after 48 h, with negative results in the G test, ruling out Candida infection. Dry, biting agar-like colonies grew on blood agar after 3 days. After 5 days, the colonies become wrinkled and stacked like leather, with velvety aerial mycelia on the surface (Figure 2B). Upon sequencing of the 16S rRNA gene, exhibited a 99.9% nucleotide homology with *N. cyriacigeorgica* (NR117334.1) in the NCBI database. These sequences were submitted to the SRA database at NCBI with the accession number PP178562.

Considering the possibility of infection related to immunosuppression, cyclophosphamide and methylprednisolone were discontinued while retaining traditional Chinese medicine, tacrolimus and pyridostigmine bromide. Additionally, the patient was prescribed trimethoprim–sulfamethoxazole (TMP-SMX; 2 tablets bid) for the treatment of *N. cyriacigeorgica* infection. After 7 weeks of treatment for MG, the patient reported slight improvement in bilateral upper eyelid ptosis, reduced fatigue during swallowing, alleviated limb weakness, reduced neck fatigue, and improved shortness of breath. After 1 month of standardized treatment for *N. cyriacigeorgica* infection, on December 1, 2021, the inflammatory indicators in the routine blood tests showed a significant decrease (WBC 7.25 × 109/L, 78.4% neutrophils, 16.4% lymphocytes, and CRP 16.9 mg/L). Chest CT showed a localized solid lesion in the basal segment of the lower lobe of the left lung, which was less extensive than before (Figures 1C,D). Bilateral drooping eyelids, shortness of breath, weakness in chewing, and weakness in both lower limbs improved. The absolute score for MG was 6 points, and the treatment outcome was judged as essentially cured. The clinical symptoms of MG and pulmonary *Nocardia* infection had improved, and it was agreed to discharge the patient. Until now, there has been no recurrence of the *Nocardia* infection in the lungs.

## Discussion

MG is an autoimmune disorder characterized by impaired neuromuscular transmission mediated by autoantibodies. Patients are categorized into early-onset (≥18 and <50 years), late-onset (>50 years) and very late-onset MG (≥65 years) (6). Skeletal muscles throughout the body can be affected, manifesting as fluctuating weakness and fatigability, with symptoms showing a pattern of worsening in the morning and improving in the evening, they are exacerbated by activity and alleviated by rest. The genus *Nocardia* is a group of aerobic actinomycetes, Gram-positive bacteria, with some species exhibiting weak acid-fast staining. They are commonly found in soil, rotten plants and dust particles (2). The disease caused by *Nocardia* mainly manifests in three forms: pulmonary infection (the most common), disseminated infection, and primary cutaneous and soft tissue infections (7). Nocardiosis is prevalent among individuals with immunodeficiency or long-term use of immunosuppressive agents.

Research shows that patients with MG often experience concomitant infections due to the combined effects of compromised autoimmune regulation, progressive deterioration of myasthenia, and long-term use of immunosuppressants and hormones (8, 9). Muralidhar Reddy et al. conducted a statistical analysis on 21 cases of nocardiosis in MG patients, identifying risk factors for *Nocardia* infection, including elderly men, thymoma, immunosuppressive therapy, and pre-existing pulmonary diseases (4). *Nocardia farcinica* and *Nocardia asteroides* were more common pathogens (4). The investigation into the etiology of *N. cyriacigeorgica* infection in the patient reveals several key points. The patient in this case was an older man working in agriculture. It is possible that he inhaled *Nocardia* while working in the fields, initially resulting in colonization throughout the body. MG can induce respiratory muscle weakness, leading to symptoms such as respiratory distress, weak coughing, dyspnea, and dysphagia, which impede the clearance of pathogens. Systemic hormone therapy, especially when used in conjunction with other immunosuppressants, compromises the immune system. Misra et al. indicated that 88% of patients exhibit comorbidities, notably prevalent in the late-onset group, with cardiovascular diseases being common (10). Our patient had multiple comorbidities, including type 2 diabetes mellitus and a history of coronary artery stenting, which exacerbated the risk of infection.

*Nocardiosis* has three primary forms: primary cutaneous infection, pulmonary infection, and disseminated infection (1, 11). It can also present as central nervous system infections, eye infections, osteomyelitis, and septic arthritis (7). *Nocardia* infections are commonly seen in immunocompromised hosts undergoing immunosuppressive therapy, such as in cases of prolonged corticosteroid use, malignancies, organ transplants, and HIV. They can also occur in non-immunocompromised hosts, primarily those with structural lung diseases like cystic fibrosis and bronchiectasis (2). We have searched for case reports and case series of *N. cyriacigeorgica* pulmonary infection during 2010-2024 in PubMed using the key words “(pulmonary) AND (*Nocardia cyriacigeorgica*).” Our search yielded 35 case reports that are summarized in Table 1. The total number of patients was 36, with an equal distribution of 18 males and 18 females (50%). Among them, 27 patients (75%) were over the age of 50. *Nocardia* pulmonary infections are commonly seen in immunosuppressed patients and those with underlying pulmonary conditions. Our statistics revealed that 9 patients (25%) had underlying pulmonary diseases, and 21 patients (58%) were immunosuppressed. Among the immunosuppressed, 6 patients (17%) had a history of cancer, and 5 patients (14%) had autoimmune diseases. Moreover, we found that 5 patients (14%) had diabetes, and 5 patients (14%) were smokers. Regarding treatment, a significant number of patients, 28 (78%), were treated with TMP-SMX, and 28 patients (78%) showed a good recovery from their Nocardial lung infection symptoms.

**Table 1:** Case reports and case series of *N. cyriacigeorgica* pulmonary infection during 2010-2024 in PubMed.

Reference	Country/Age/Gender	Predisposing factors	Treatment	Outcome	
Akcaglar, 2008	Turkey/37/M	Basedow–Graves disease, diabetes	TMP–SMX, amphotericin B, vancomycin, imipenem	Died	(20)
Cargill, 2010	United Kingdom/85/F	History of ischaemic heart disease, chronic obstructive pulmonary disease and polymyalgia rheumatica	Piperacillin/tazobactam, vancomycin, imipenem, doxycycline	Died	(21)
Chavez, 2011	United States of America/58/M	Obesity, hypertension, and diet-controlled type 2 diabetes mellitus	TMP–SMX	Recover well	(22)
Hadano, 2011	Japan/82/F	Colectomy for colon cancer history	TMP–SMX	Recover well	(23)
Madden, 2012	United States of America/9/M	Chronic granulomatous disease	TMP-SMX, linezolid, levofloxacin, voriconazole	Recover well	(24)
Lavalard, 2013	France/71/F	History included a diagnosed Waldenström's disease 20 years earlier, treated with cytotoxics, mitral and aortic insufficiency, excision of a frontal basal cell epithelioma, labial herpes, dry mouth and eyes without etiology and active smoking	TMP-SMX, rifampicin	Died	(25)
Severo, 2013	Brazil/77/F	Bronchiectasis, chronic obstructive pulmonary disease	NA	NA	(26)
Yagi, 2014	Japan/72/M; Japan/53/F	Rheumatoid arthritis, untreated *Mycobacterium avium* complex lung disease; 6-pack-year smoking history, a history of *Mycobacterium avium* complex lung disease	TMP–SMX; NA	Recover well; NA	(27)
Eshraghi, 2014	Iran/55/F	Kidney transplant	Trimoxazole, ceftriaxone	Recover well	(28)
Berring, 2014; Garcia,2015	Pakistan/6/M	Nephrotic syndrome	TMP-SMX, linezolid	Recover well	(29)
	United States of America/77/M	Myasthenia gravis, history of treated pulmonary tuberculosis	TMP-SMX, meropenem, linezolid	Recover well	(5)
Castellana, 2016	Italy/75/M	Chronic Obstructive Pulmonary Disease, smoking history	TMP–SMX	Recover well	(30)
Trastoy, 2017	Spain/81/F	Non-Hodgkin B-cell lymphoma and diabetes mellitus	TMP–SMX, meropenem, voriconazole (MI)	NA	(31)
Rivera, 2017	Puerto Rico/60/M	NA	TMP–SMX	Recover well	(32)
Vignesh, 2017	India/1M	Recurrent bloody stools, cervical and inguinal lymphadenitis, bilateral peri broncho vascular interstitial thickening with patchy consolidation in the left lower lobe, diffuse colon edema, polyclonal hypergammaglobulinemia	Mesalamine, anti-tubercular therapy (isoniazid, rifampicin, and ethambutol)	NA	(33)
Wu, 2018	China/55/F	Allergic bronchopulmonary aspergillosis	TMP–SMX and meropenem	Recover well	(34)
Kobayashi, 2018	Japan/52/F	Olfactory neuroblastoma	TMP-SMX	Recover well	(35)
Mahajan, 2019	United States of America/70/M	Low-dose prednisone, decreased appetite, proximal lower extremity weakness and leg spasms	TMP-SMX, meropenem, linezolid	Died	(36)
Karan, 2019	Serbia/70/M	Epileptic	Sulfamethoxazole and ceftriaxone	Recover well	(37)
AlMogbel, 2020	Saudi Arabias/65/F	End-stage renal disease, relied on hemodialysis, and suffered from ischemic heart disease, idiopathic thrombocytopenia and hypothyroidism	Vancomycin and ceftriaxone	Recover well	(38)
Yu, 2020	China/79/M	Membranous kidney disease	TMP-SMX, linezolid, methylprednisolone, and meropenem	Recover well	(39)
Saha, 2020	United States of America/72/M	Chronic obstructive pulmonary disease	TMP-SMX	Recover well	(40)
Lin, 2021	China/37/F	Chronic granulomatous disease	TMP–SMX	Recover well	(41)
Browne, 2021	Afghani/77/M	Diabetes, extensive history of chewing tobacco use	TMP–SMX, ceftriaxone and dexamethasone	Recover well	(42)
Colaneri, 2021	Italy/45/F	Human immunodeficiency virus infection infection	TMP/SMX, linezolid, and amikacin (MI)	Recover well	(43)
Tsuchiya, 2022	Japan/61/M	Diabetes, bronchiectasis	TMP–SMX	Recover well	(44)
Roy, 2022	United States of America/39M	Systemic lupus erythematosus	TMP–SMX, amikacin, voriconazole (MI)	Recover well	(45)
Kobashi, 2022	Japan/61/F	NA	TMP–SMX, meropenem, clarithromycin (MI)	Recover well	(46)
Bejcek, 2022	Hispanic/67/M	Non–small cell lung cancer	TMP–SMX	Recover well	(47)
Hong, 2023	China/55/F	CD4+ T cell deficiency	TMP–SMX	Recover well	(48)
Ye, 2023	China/63/F	Smoking history of over 40 years	TMP–SMX and voriconazole (MI)	Recover well	(49)
Gandham, 2023	India/41/M	Post-transplant	TMP–SMX	Recover well	(50)
Urbantat, 2023	Germany/14/F	Primary ciliary dyskinesia	TMP-SMX, meropenem	Recover well	(51)
Zhang, 2023	China/78/F	NA	Meropenem, linezolid, Sulfamethoxazole	Recover well	(52)
Bove, 2024	DOM/31/F	NA	TMP-SMX	Recover well	(53)

M, male; F, female; TMP–SMX, trimethoprim-sulfamethoxazole; MI. Mixed infections.

On the basis of typical clinical manifestations such as fluctuating muscle weakness, diagnosis can be made by meeting any of the following three criteria: pharmacological examinations, electrophysiological characteristics, and serum anti-acetylcholinesterase receptor or other antibody detection. The clinical manifestations and pulmonary imaging of pulmonary nocardiosis lack specificity, posing challenges for early diagnosis. In the present case, the patient's sputum smear exhibited morphological features highly suggestive of *Nocardia* infection under the microscope. Combined with the slow growth characteristics of *Nocardia* species, it was cultured successfully through extending the incubation period. Therefore, for slow-growing bacteria like *Nocardia*, direct smear staining microscopy and prolonged incubation time are crucial.

Currently, the treatment of MG primarily revolves around cholinesterase inhibitors, glucocorticoids, immunosuppressants, intravenous immunoglobulin, plasmapheresis, and thymectomy (12). Many studies have indicated that some Chinese medical approaches demonstrate promising outcomes in managing MG. Integrating Chinese and western medical practices can reduce the recurrence rate of the disease, minimize adverse reactions and complications, as well as modulate the immune function (13–16). The relative severity of MG in patients is reflected by the absolute score of clinical evaluation, which assesses the degree of muscle weakness and fatigue without necessitating any instrumentation. Scoring ranges from 0 to 60 points, with higher scores indicating more severe symptoms. The approach utilized in this case involved integrating both traditional Chinese medicine and western medicine to treat severe MG. Upon admission, the absolute score was 31, which significantly decreased to 6 following treatment, indicating remarkable therapeutic efficacy. TMP–SMX is the preferred treatment for nocardiosis, with monotherapy often used for nonsevere cases. Empiric multidrug therapy (such as carbapenems, TMP–SMX, amikacin, linezolid, or parenteral cephalosporins) is recommended for severe pulmonary infections, disseminated infections, and central nervous system infections (17, 18). Sulfonamide–carbapenem combination therapy is used empirically for nocardiosis, but all *Nocardia* isolates should ideally be identified to the species level and subjected to susceptibility testing to guide optimal treatment. Patients with localized pulmonary infections should receive treatment for >6 months, while those with complicated or disseminated diseases should undergo antibacterial therapy for >9 months (18, 19). The present case utilized the TMP–SMX for anti-infective therapy. The significant decrease in inflammatory markers and improvement in clinical symptoms before and after treatment suggested a pronounced therapeutic effect. Based on the comprehensive assessment, the clinical symptoms of MG and pulmonary *Nocardia* infection improved, and reached the criteria for clinical cure. Therefore, the patient was approved for discharge. Until now, there has been no recurrence of the Nocardia infection in the lungs.

## Conclusion

To our knowledge, cases of coexisting MG and *Nocardia* infection are relatively rare. This present case represents the second instance, which was aimed at further elucidating the relationship between MG and pulmonary *Nocardia* infection by adding another clinical example. It underscores the heightened risk of developing pulmonary Nocardiosis following the use of hormone and immunosuppressive agents in MG patients. Clinicians should be cognizant of this and consider the possibility of *Nocardia* infection when conventional empirical treatments fail, thereby facilitating timely communication with microbiology laboratories to appropriately extend culture duration and prevent misdiagnosis.

## Data Availability

The original contributions presented in the study are included in the article/supplementary material, further inquiries can be directed to the corresponding authors.
